# Trypanosomiasis Control, Democratic Republic of Congo, 1993–2003

**DOI:** 10.3201/eid1109.041020

**Published:** 2005-09

**Authors:** Pascal Lutumba, Jo Robays, Constantin Miaka mia Bilenge, Victor Kande Betu Ku Mesu, Didier Molisho, Johan Declercq, Wim Van der Veken, Filip Meheus, Jean Jannin, Marleen Boelaert

**Affiliations:** * Programme National de Lutte contre la Trypanosomiase Humaine Africaine, Kinshasa, Democratic Republic of Congo;; † Institute of Tropical Medicine, Antwerp, Belgium;; ‡ Fonds Médical Tropical, Kinshasa, Democratic Republic of Congo;; § Coopération Technique Belge, Kinshasa, Democratic Republic of Congo;; ¶ Institute of Development Policy and Management, Antwerp, Belgium;; # World Health Organization, Geneva, Switzerland

**Keywords:** human African trypanosomiasis, Democratic Republic of Congo, disease control, epidemiology, evaluation, efficiency, effectiveness, research

## Abstract

In the Democratic Republic of Congo (DRC), human African trypanosomiasis (HAT) reached unprecedented levels in the 1990s. To assess recent trends and evaluate control efforts, we analyzed epidemiologic and financial data collected by all agencies involved in HAT control in DRC from 1993 to 2003. Funds allocated to control populations, as well as to the population screened, doubled from 1993 to 1997 and from 1998 to 2003. The number of cases detected decreased from 26,000 new cases per year in 1998 to 11,000 in 2003. Our analysis shows that HAT control in DRC is almost completely dependent on international aid and that sudden withdrawal of such aid in 1990 had a long-lasting effect. Since 1998, control efforts intensified because of renewed donor interest, including a public-private partnership, and this effort led to a major reduction in HAT incidence. To avoid reemergence of this disease, such efforts should be sustained.

## Abstract 2

Efforts to control human trypanosomiasis, which sharply reduced the disease, must be sustained.

Human African trypanosomiasis (HAT), or sleeping sickness, is a vectorborne disease caused by the parasite *Trypanosoma brucei*. East African HAT is caused by *T. b. rhodesiense* and West African HAT, the subject of this article, by *T*. *b*. *gambiense*. The latter species causes a slowly progressing fatal disease with few specific symptoms or none in its initial stage (1). The only proven effective way to control *T*.*b*. *gambiense* HAT is mass population screening and treatment of those infected. Well conducted campaigns reduce the human parasite reservoir and therefore HAT incidence (2).

Substantial observational evidence from Sudan (3), Uganda (4), Equatorial Guinea (5), and the Bandundu region in former Zaire (6) has shown that intensive screen-and-treat programs effectively reduce HAT incidence. So far, no evidence has shown that adding vector control to active case finding is effective, and vector-control efforts are limited (5). HAT is one of the so-called neglected diseases that afflict the developing world; the term indicates the lack of drug research and development for these conditions (7). In the field of HAT, the situation was so bleak by 1998 that production of sleeping sickness drugs was no longer guaranteed. A public-private partnership was established in 2001 between the World Health Organization (WHO) and Sanofi-Aventis (Paris, France), the main pharmaceutical manufacturer of anti-HAT drugs. Sanofi-Aventis donated the 3 most used anti-HAT drugs (DFMO [difluoromethylornithine], melarsoprol, and pentamidine) for 5 years and also offered funding for disease control and innovative research. Bayer AG (Leverkusen, Germany) has donated a 5-year supply of suramin, another anti-HAT drug. These donations were welcomed by HAT control programs, which used to spend up to 46% of their annual budgets on the purchase of drugs (S. Van Nieuwenhove et al., unpub. data).

The sustainability of HAT control has been a recurrent concern, as exemplified by the postcolonial history of sleeping sickness control in the Democratic Republic of Congo (DRC). By 1960, the year of DRC's independence, HAT was almost completely eliminated, but by 1976, many new cases were diagnosed. HAT control received substantial international aid during the 1980s, which amounted to >90% of DRC's HAT budget. However, this international support was suddenly withdrawn after the massacre of students at the Lubumbashi University in May 1990 (8). Inevitably, sleeping sickness returned to DRC in full measure. In 1994, donors again allocated financial support for HAT control as humanitarian emergency aid and channeled its implementation through nongovernmental organizations. However, by 1997, the epidemiologic situation seemed little better than in the 1930s (9,10) and showed a rising trend that was cause for concern. Moreover, the HAT problem in DRC was no longer restricted to remote rural districts: urban areas such as Kinshasa were reporting cases (11). In 1998, Belgian bilateral aid for HAT resumed under a 5-year support program, and full screening and treatment programs were restarted. Several authors ascribe the reemergence of HAT in DRC primarily to the interruption of bilateral and multilateral aid that occurred after 1990 (12–14). The drastic reduction in specific control activities at a time when the epidemic was spreading, in the context of overall collapse of the Congolese health infrastructure, most likely contributed to the exponential rise in HAT cases after 1990. We examined the recent trends of HAT in DRC and evaluated the effects and sustainability of the control program.

## Methods

### Context

DRC has a surface area of 2.345.000 km^2^ and ≈60 million inhabitants, for a density of 25 inhabitants/km^2^. Administratively, DRC is subdivided into 11 provinces, and HAT is endemic in 9 of them (Programme National de Lutte contre la Trypanosomiase Humaine Africaine [PNLTHA], unpub. data). Since 1990, the country has been devastated by political turmoil and civil war (1996–1997 and 1998–2003). The health status of the population has deteriorated because of progressive breakdown of health infrastructures, disease outbreaks, and the reemergence of endemic diseases such as tuberculosis and HAT. The emergence of HIV/AIDS has added to this catalog of health disasters.

**Figure 1:**
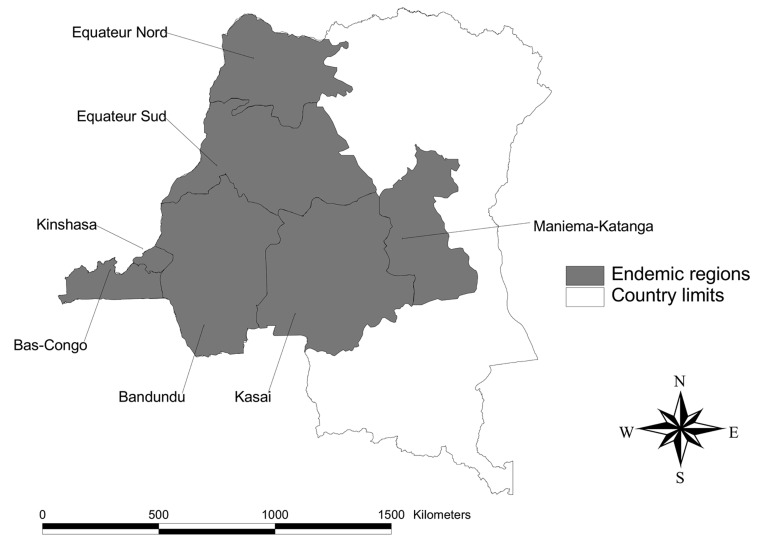
Disease-endemic regions (indicated by shaded areas) in the Democratic Republic of Congo, as managed by human African trypanosomiasis program.

HAT control in DRC is organized by a national program, PNLTHA. This program divides HAT-endemic areas into 7 regions, each under the responsibility of a regional coordinator (Figure 1). These regions do not coincide with the administrative divisions (provinces).

The main control strategy of PNLTHA is to actively screen the population at risk by specialized mobile teams (15), who refer patients with confirmed cases to regular health services and specialized centers for treatment. Screening was based on the palpation of cervical glands until 1996, when a serologic screening test (card agglutination test for trypanosomiasis [CATT]) was added to the algorithm (16). Each mobile team screens ≈40,000 persons/year. A considerable amount of passive case finding takes place as well, for example, when the regular health service staff diagnose HAT in a patient who arrives for a consultation. PNLTHA's control strategies also include vector control.

### Data Sources

We used the PNLTHA epidemiologic surveillance database that included all HAT cases detected by mobile teams and regular health services since 1926. For 1993–2003, we examined the monthly reports compiled by the regional PNLTHA coordinators, with the exception of those from Maniema-Katanga and the Province Orientale because they were incomplete and fragmentary as a consequence of the ongoing war.

We distinguish 2 discrete periods for the analysis of international aid. From 1993 to 1997, only humanitarian aid budgets were allocated to HAT control, typically lasting for a maximum of 6 months. Because of the political turmoil at that time in DRC, international aid for HAT was given as "indirect aid," i.e., donors would give cash grants to 3 nongovernmental organizations (NGOs)—Fonds Médical Tropical (FOMETRO), Medische Missie Samenwerking (MEMISA), and Médecins sans Frontières (MSF)—and rely on them for implementation. MEMISA and MSF would supplement this indirect aid with funds they had privately raised. Between 1998 and 2003, the Congolese government again benefitted from long-term international aid programs, and the Belgian Technical Cooperation (BTC) launched its own technical assistance program for HAT control. The same NGOs continued to play a major role in implementation, as well as partly funding, these control activities. For the period under study, WHO funds were donated directly to PNLTHA, while those from the European Union were given to FOMETRO.

We obtained financial data on budgets and expenditure for HAT control directly from the various donor agencies and cross-checked data with all the implementing agencies (PNLTHA, the 3 NGOs, and the BTC engaged in HAT control in DRC during the period under study) (Table 1). Only funds allocated to HAT control were incorporated in our study; we excluded funds earmarked for research. To avoid duplication, we categorized financial resources by donating and not by implementing agency. Over the entire study period, the Congolese government only allocated funds for personnel costs, and those were included in our computations at an average salary of US $12.50 per month per person. All international aid was donated in cash directly to the NGOs or BTC.

**Table 1:** Financial resources converted to constant 1998 US$ and their origin during the first (1993–1997) and the second period (1998–2003)*†

	Type of donor	1993–1997		1998–2003	
		US$	Percentage	US$	Percentage
Belgian government	Bilateral	4,508,774	69.2	14,566,002	87.1
European union	Bilateral	1,337,946	20.5	656,367	3.9
Congolese government	NA	270,611	4.2	329,441	2.0
WHO	Multilateral	0	0.0	527,698	3.2
Pain pour le Monde‡	NGO	70,430	1.1	68,411	0.4
MSF‡	NGO	0	0.0	104,233	0.6
MEMISA‡	NGO	70,965	1.1	462,906	2.8
AFRICA‡	NGO	0	0.0	6,440	0.1
Caritas–Germany‡	NGO	254,506	3.9	0	0.0
Total		6,513,232	100.0	16,721,496	100.0
Total per year		1,302,646		2,786,916	

*NA., not applicable; NGO, nongovernmental organization; WHO, World Health Organization; MSF, Médecins Sans Frontières Belgique; MEMISA, Medische Missie Samenwerking; AFRICA, Association des Femmes pour les Rencontres Intellectuelles et Culturelles en Afrique. †Sanofi-Aventis/Bayer in-kind drug donation not included; see text. ‡The amounts mentioned for NGOs are limited to the "own funds," i.e., funds that they had privately raised and spent on sleeping sickness control. Several NGOs were implementing HAT control activities with funds provided by the bilateral or multilateral donors.

Expenditure in Belgian francs was converted into US dollars, according to the exchange rate that applied at time of expenditure. Expenditure in euros was converted at a fixed rate of 40.3399 Belgian francs = 1 euro. The exchange rate between the US dollar and euro was the average exchange rate per year based on Federal Reserve Statistical Release (available at http://www.federalreserve.gov/releases/H10/Hist/). All current dollars were converted to constant 1998 dollars by using the US Office of Labor Consumer price Index. All data were stored and analyzed in an Excel database (Microsoft Corp., Redmond, WA, USA).

### Definitions

PNLTHA defines a new HAT patient as a person whose condition has, for the first time, been diagnosed by parasitologic examination as sleeping sickness. Relapse cases are thus not included in this study. The HAT detection rate is the number of newly detected cases, expressed as a proportion of the screened population. We distinguish the active detection rate (ADR), in which data are collected through active case finding, from the overall detection rate, which also includes cases detected at health facilities. The coverage rate of the population is the proportion of the population tested (through active or passive case finding) divided by the population at risk for HAT. The participation rate applies only to active case finding and is defined as the number of persons screened by the mobile teams divided by the target population. The proportion of treated patients is the number of persons who received HAT treatment divided by the number of persons detected with HAT.

### Evaluation Method

We structured our evaluation of the HAT control program in the form of input, process, output, and outcome analyses (17) and according to the method of Bouchet et al. (18) (Table 2). Input represents the human and financial resources invested in the program. Drug availability, measured as the number of occasions that the stock ran out during the period under study, was also considered as an input. Process indicators are not reported in this study because they are relevant only to the daily management of the program. Program output was measured through an analysis of coverage of the population at risk, the participation rate in screening, the number of detected HAT cases, the proportion of patients with detected HAT patients who received treatment, and the proportion of patients with treated cases that have been followed-up correctly. We report the annual HAT detection rate, both nationally and for each region, as indicators of program outcome.

**Table 2:** Indicators for evaluating population screening for human African trypanosomiasis (HAT)*

Indicators	
Input	Financial resources Human resources Availability of tests and anti-HAT drugs
Process	Identification of villages at risk Census of population at risk Involvement of population at risk in active case detection Lymph node palpation CATT test Parasitologic test for HAT confirmation Lumbar puncture to determine the stage of disease Treatment Treatment follow-up
Output	Coverage rate of population at risk Participation rate Identification of suspects Identification of HAT cases Proportion of HAT cases detected and treated Proportion of HAT cases treated and followed
Outcome	Annual HAT detection rate and trend

*CATT, card agglutination test for trypanosomiasis; LN, lymph node palpation.

## Results

### Outcome

**Figure 2:**
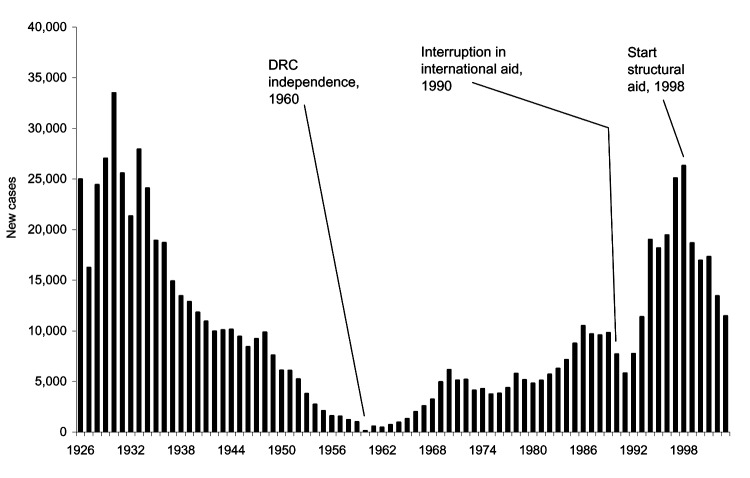
Number of new human African trypanosomiasis new cases in the Democratic Republic of Congo, 1926–2003.

Figure 2 represents the evolution in the annual number of newly detected HAT cases in DRC from 1926 to 2003. Between 1960 and 1989, the figure shows an increasing trend with 2 small peaks in 1970 and 1986. This trend was interrupted in 1990 and 1991, which coincides with the sudden arrest of control activities in 1990. When humanitarian aid was launched in 1993, the annual number of detected cases increased markedly. The peak was reached in 1998, when the control program detected 26,318 new HAT cases in a screened population of 1,472,674 persons.

**Figure 3:**
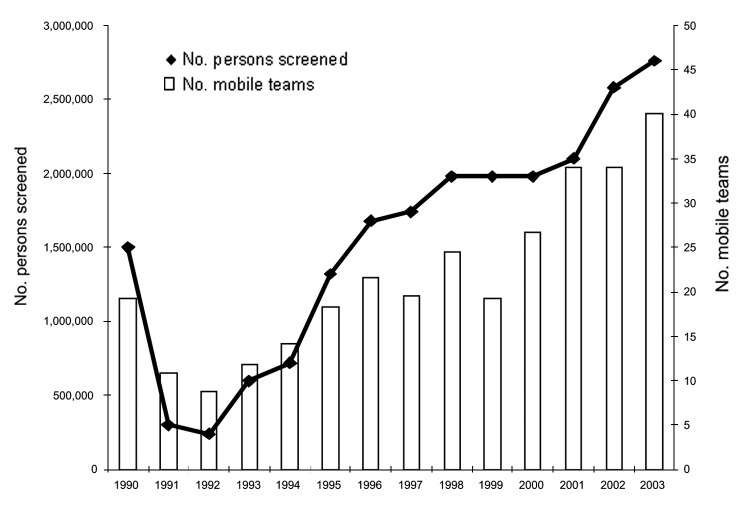
Population screened per year and number of mobile teams operating in the Democratic Republic of Congo, 1990–2003.

**Figure 4:**
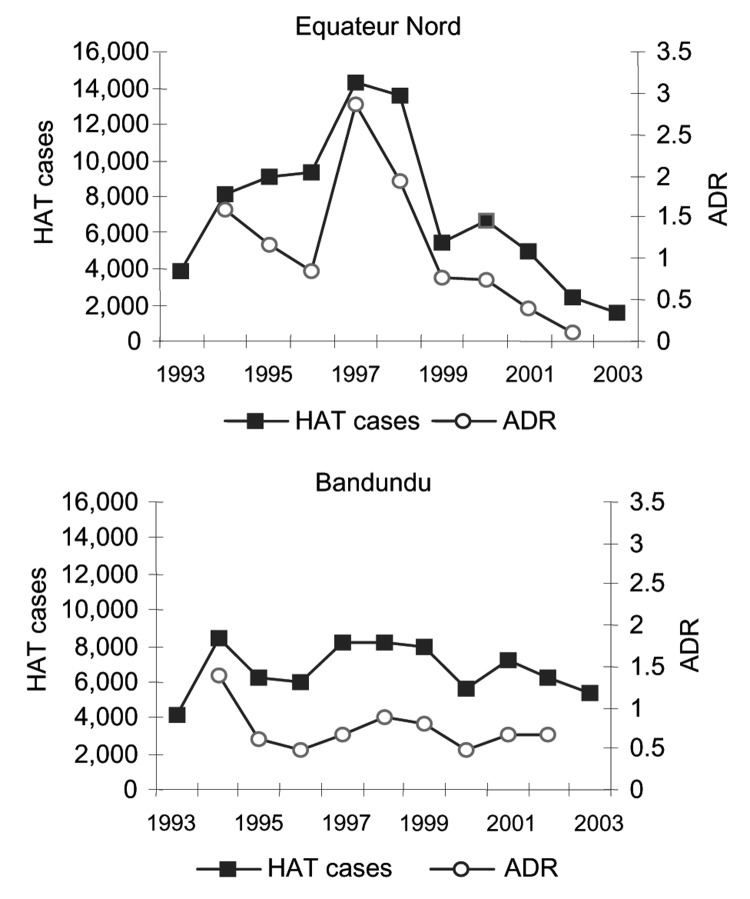
Number of new human African trypanosomiasis cases and active detection rate (ADR), by region, Democratic Republic of Congo,1993–2003.

After 1998, a marked decline occurred in the number of HAT cases detected, which was not due to an overall decrease in screening activities; the number of operational mobile teams and number of screened persons continued to increase over that period (Figure 3). The overall HAT detection rate, based on active case finding, declined from 1.1% in 1994 to 0.3% in 2002. However, this overall decline in detected HAT cases masks differences between regions. Figure 4 shows the evolution of the number of HAT cases and active detection rate, by region, from 1993 until 2002.

### Input

In 1990, 25 mobile teams covered the population at risk. With the reduction in external financial support, 10 teams remained operational from 1991 to 1993. This number slowly increased to 33 teams in 1998 and to 46 teams in 2002. These increases were concentrated in the regions of Bandundu, Equateur-Nord, and Kasai, where the number of teams rose to 13, 13, and 7, respectively, which accounts for 33 mobile teams of 46. In 1993, PNLTHA staff was 250. This number increased progressively to 580 in 2001 and remained stable at 580 in 2003.

From 1993 to 1997, total annual expenditure amounted to US$1,302,646, while in the period 1998–2003, total annual expenditure doubled to US$2,786,916. Table 1 shows the amount and the origin of the financial resources. The budget breakdown was as follows: functioning costs (fuel, vehicle maintenance, supervision, training, stationery, etc.) (32.0%), personnel time (26.4%), HAT drugs (24.3%), laboratory reagents (7.3%), and other material and equipment (10%). The average expenditure per HAT case detected and treated is shown in Table 3.

**Table 3:** Average expense per examined person and average cost per case detected and treated (converted to constant 1998 US$) in active case finding*

	1993–1997	1998–2003
No. screened in population at risk	5,003,477	10,659,210
No. HAT cases detected and treated†	41,500	55,500
Average expenditure per case screened (US$)	1.30	1.57
Average expenditure per case detected and treated (US$)	156.9	301.2

*HAT, human African trypanosomiasis. †Takes into account only active case finding by mobile teams and the assumption of a treatment rate of 100%.

From 1993 to 2001, implementing agencies spent, on average, 24% of their budgets in purchasing trypanocidal drugs (range 12%–44%). Though the Sanofi-Aventis/Bayer donation program was established in 2001, in practice, implementing agencies could continue working throughout 2002 with existing drug stocks. For 2003, a detailed analysis of amount of donated drugs versus purchased drugs consumed was not possible, and we therefore ignored the in-kind drug donation in Table 1. The full effect of the donation will only become clear after 2004, although can be estimated by its monetary value (Table 4). PNLTHA records the number of patients who have been treated by drug regimen, and these data allowed us to estimate the quantity of the trypanocidal drugs that were required, as well as the total cost of those drugs, calculated according to the preferential price, which was valid until 2001. We estimated that the total drug cost per year in DRC, for treating ≈14,000 HAT patients/year, corresponded to ≈US$600,000/year. This value is consistent with the previous estimate that 24% of budgets are reportedly used for drugs. Notably, since the public-private partnership was established in 2001, supplies of trypanocides never ran short, whereas this problem was a matter of continuous concern before.

**Table 4:** Estimated costs of anti-HAT drugs consumed in the Democratic Republic of Congo from 2001 to 2003, based on observed number of patients treated by regimen*†

Drug	Observed no. patients treated	Drug quantity/patient required	Total quantity drugs required	Unit price‡ US$	Total (US$)
Pentamidine	13,957	8	111,656	2	223,312
Suramin	2,604	6	15,624	7	109,368
Pentamidine-suramin	104	8	832	2	1,664
		6	624	7	4,368
Melarsoprol	24,456	9	220,104	7	1,540,728
Eflornithine	377	14	5,278	22	116,116
Nifurtimox§	1,168	100	116,800	0	0
Melarsoprol-nifurtimox	1,581	4	6,324	7	44,268
		64	101,184	0	0
Total	44,247				2,039,824

*HAT, human African trypanosomiasis. †Source of data: Programme National de Lutte contre la THA (PNLTHA) annual reports. The price of drugs is based on prices reported by the World Health Organization, 1998. Data for the HAT cases retreated in 2003 are not available. ‡Preferential price applicable during the period. §PNLTHA did not buy nifurtimox in this period.

### Output

The population at risk in the DRC has been estimated at 12,600,000 persons (PNLTHA, unpub. data). Screening and treatment of the at-risk population is estimated to have risen from 6% in 1993 to 19% in 2003. We observed notable differences between regions. Equateur-Nord had a coverage rate of >50%, while in the other regions the rate ranged from 10% to 20%. Figure 3 shows that the number of persons screened each year almost tripled from 1993 to 2003.

The participation rate of the population in active case finding was almost 96% in 1998. By 2002, the rate had fallen to 78%. The proportion of new patients who received treatment was ≈95% throughout the whole country but varied from region to region, from 89% to 100%. From 2001 to 2003, a total of 44,247 patients were treated with pentamidine (31.5%), suramin (5.8%), the combination pentamidine-suramin (0.2%), melarsoprol (55.2%), eflornithine (0.85%), nifurtimox (2.6%), and the combination melarsoprol-nifurtimox (3.5%) (because nifurtimox is not registered for use against HAT in DRC, it was given on a compassionate basis when no other drugs were available or when melarsoprol treatment failed).

## Discussion

After the number of cases peaked in 1998 with 26,000 new cases, the annual number of HAT cases reported in DRC has decreased to 10,900 cases in 2003. From 1993 to 2003, the annual number of persons screened for HAT, as well as financial resources allocated to HAT control in DRC, has doubled.

The increase in reported cases and in the detection rate observed between 1993 and 1997 can be attributed to increased transmission but also to renewed efforts after several months when active case finding was interrupted. However, the striking decrease in HAT cases from 1998 to 2003 cannot be explained by decreased case-detection efforts because the number of persons screened in the same period doubled. Changes in detection rates through active case finding are difficult to interpret because the population reached is not the same over time. The additional number of persons screened might come from populations that were less at risk in the first place, as happened, for example, in Ville de Kinshasa, where a new mobile team started operating in May 2001 in an area with lower prevalence. Population movements during the war could, in theory, also explain the observed changes in HAT prevalence, but no noteworthy migration from disease-endemic to disease-nonendemic areas or vice versa took place over the study period. We therefore conclude that the decreasing trend in HAT case detection observed in DRC since 1999 is real. Most likely this trend is explained by the intensification of control efforts, the steep increase in resource allocation since 1998, and a major drug donation in 2001. The systematic use of CATT as the serologic screening test in 1996 has probably contributed to a decline in transmission, because it increased screening effectiveness (15).

However, these national figures hide important differences between regions. In the northern and southern Equateur regions and in Kinshasa, the absolute number of HAT cases and detection rates has declined, whereas these indicators remain stationary in the Bas Congo, Kasai, and Bandundu regions. In fact, the decline observed at national level is, to a large extent, based on the decline observed in 1 region, Equateur-Nord, which experienced a major outbreak but brought it under control by an intensive and well-coordinated campaign.

A similar rapid decrease in the number of HAT cases has been observed by Van Nieuwenhove and Declercq (19) in southern Sudan and by Paquet et al. (4) in Uganda. However, the HAT epidemic reemerged in southern Sudan after control activities were stopped, indicating that disease control efforts should be maintained even when prevalence is low (20–22).

Our analysis showed how HAT control in DRC almost completely depends on international aid and that the interruption of financing from 1990 to 1991 had a long-lasting negative effect on case load. Funding may be discontinued for different reasons, such as changes in donor policies or priorities, so HAT control remains vulnerable. Private NGOs have so far accounted for a minor part of funding in DRC, although they played a role both in advocacy and in program implementation. The recent public-private alliance with pharmaceutical companies not only made continued care for HAT patients possible again but also released substantial financial resources that can be used in the future for operations in DRC. Moreover, through direct financial support to research, training, and rehabilitation, the public-private partnership has contributed to a wider alliance and extension of activities. However, the fact that the 3 main drugs used to treat HAT patients are produced and donated by a single company creates a new type of dependency. Care for HAT patients may be seriously compromised if production or donation stopped for any reason, for example, a company takeover, management changes, or a change in the company's priorities.

The disparities now emerging in disease epidemiology in different parts of DRC call for the adoption of differential control strategies in different regions of the country. Where the ADR has dropped to low levels, screening intervals could be lengthened. Alternatively, and with lower cost, surveillance methods could be used that detect emerging epidemics at an early stage, such as serologic surveys, or that rely on data collection from passive case finding and enhanced diagnosis in the primary health structures (23). Where ADR remains high, the program must identify the reasons for this and find solutions to make control more effective. Furthermore, the increase in treatment failures in the southeastern part of the country should be carefully monitored, and evolving parasite resistance should be thoroughly investigated.

Our analysis shows that successful HAT control is possible, but that it depends on continued financial support and drug availability. Therefore, the governments of disease-endemic countries and the international community must make long-term financial commitments to ensure the continuity of HAT control activities. This necessitates sound financial sustainability planning for HAT control, as is already done, for instance, in childhood immunization (24). Research is necessary on how to rationalize control activities so that control programs can adopt the most effective and efficient strategies.
